# CytoConverter: a web-based tool to convert karyotypes to genomic coordinates

**DOI:** 10.1186/s12859-019-3062-4

**Published:** 2019-09-11

**Authors:** Janet Wang, Thomas LaFramboise

**Affiliations:** Department of Genetics and Genome Sciences, Case Western Reserve University, Cleveland, OH 44106 USA

**Keywords:** Cytogenetics, Chromosomal abnormalities, Karyotypes, Text parsing

## Abstract

**Background:**

Cytogenetic nomenclature is used to describe chromosomal aberrations (or lack thereof) in a collection of cells, referred to as the cells’ karyotype. The nomenclature identifies locations on chromosomes using a system of cytogenetic bands, each with a unique name and region on a chromosome. Each band is microscopically visible after staining, and encompasses a large portion of the chromosome. More modern analyses employ genomic coordinates, which precisely specify a chromosomal location according to its distance from the end of the chromosome. Currently, there is no tool to convert cytogenetic nomenclature into genomic coordinates. Since locations of genes and other genomic features are usually specified by genomic coordinates, a conversion tool will facilitate the identification of the features that are harbored in the regions of chromosomal gain and loss that are implied by a karyotype.

**Results:**

Our tool, termed CytoConverter, takes as input either a single karyotype or a file consisting of multiple karyotypes from several individuals. All net chromosomal gains and losses implied by the karyotype are returned in standard genomic coordinates, along with the numbers of cells harboring each aberration if included in the input. CytoConverter also returns graphical output detailing areas of gains and losses of chromosomes and chromosomal segments.

**Conclusions:**

CytoConverter is available as a web-based application at https://jxw773.shinyapps.io/Cytogenetic__software/ and as an R script at https://sourceforge.net/projects/cytoconverter/. Supplemental Material detailing the underlying algorithms is available.

**Electronic supplementary material:**

The online version of this article (10.1186/s12859-019-3062-4) contains supplementary material, which is available to authorized users.

## Background

Many human diseases, particularly cancer, are caused by or driven by gains and losses of chromosomes or chromosomal segments [2]. In cancer, oncogenes are often found within regions of gain, while tumor suppressor genes are frequently deleted [13]. Chromosomal abnormalities also cause many known syndromes, and may be suspected as causes of undiagnosed diseases [14]. As such, testing for gains and losses is an important component of both research and clinical practice.

Before the advent of higher-resolution techniques, karyotypes were the primary method used to characterize chromosomal aberrations, and are still widely used. A karyotype summarizes the state of the genetic material in a collection of cells. The naming system for describing chromosomal changes – cytogenetic nomenclature – is dictated by the International System for Human Cytogenomic Nomenclature (ISCN) [11]. It describes the total number of chromosomes in the cell, the unmodified sex chromosomes, and the chromosome(s) with abnormalities present. Karyotyping relies on the use of banding techniques, which have allowed researchers to describe the locations of microscopic-level changes such as translocations and band-sized or larger deletions and duplications. The completion of the human genome sequence in the early 2000s, however, enabled description of chromosomal aberrations in terms of genomic coordinates, which can specify locations at nucleotide-level resolution [12]. Technological advances such as genotyping microarrays (reviewed in LaFramboise, 2009 [8]) and, more recently, next-generation sequencing (NGS) have spurred the community to more commonly frame deletions and gains in terms of genomic coordinates. However, karyotyping is still used today [9]. The use of cytogenetics remains popular for analyzing blood samples [5], for example. Additionally, there is copious archival data (e.g. the Mitelman Database of Chromosome Aberrations and Gene Fusions in Cancer) recorded in cytogenetic nomenclature. Large numbers of these karyotyped samples have not been subjected to microarray or NGS-based analyses, and in many cases the DNA is no longer available. In order to allow researchers to more easily identify the genomic entities such as genes and regulatory loci that are harbored in the gained and lost regions of karyotyped samples, we have developed a converter that parses the karyotypes and returns the corresponding gains and losses in terms of genomic coordinates.

A handful of tools are currently available to computationally analyze cytogenetic nomenclature. CyDAS [7] can characterize the loss and gain of chromosome material and can create karyograms from cytogenetic nomenclature. However, the program does not appear to have updated since 2004, and gives information using band locations, not genomic coordinates. The National Center for Biotechnology Information (NCBI) provides a tool to convert cytogenetic bands into genomic coordinates. However, this tool does not characterize losses and gains, and only converts individual bands and not karyotypes (https://www.ncbi.nlm.nih.gov/genome/tools/cyto_convert/).

### Implementation

CytoConverter is written in R. It accepts single karyotype strings and/or text, tab-delimited tables as input. An input table must have two columns, one indicating sample names and the other containing the karyotypes. The rules of cytogenetic nomenclature should be followed according to the ISCN 2016. CytoConverter outputs gains and losses, labelled by sample name, in hg18, hg19, or GRCh38 coordinates. If cell count information is provided in the input karyotype, CytoConverter also reports the numbers of cells harboring each aberration, and the total number of cells in the sample.

**Fig. 1:**
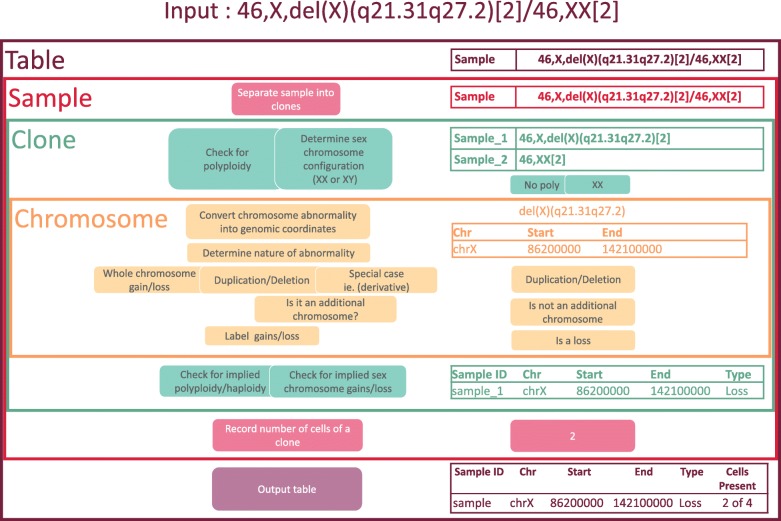
Summary of CytoConverter implementation. This figure walks through the program’s parsing of the example input karyotype 46,X,del(X)(q.21.31q27.2[2]/46,XX [2]

More specifically, CytoConverter divides a karyotype into individual clones, where applicable, reporting results according to each clone’s karyotype. It will output a table of losses and gains, with each row consisting of the sample name (adding “_n” to the sample name for the *n*^th^ clone in the karyotype, if multiple clones are present), chromosome number, beginning base position, ending base position, whether the region was lost or gained, and number of cells in the clone out of total number of cells in the sample if cell number information is provided in the input karyotype. CytoConverter also provides graphical output displaying a heatmap in which rows represent distinct samples or clones and columns represent chromosomal locations. Gains are displayed in red, hemizygous losses in blue, and homozygous losses in yellow. An overview of the algorithm used to parse cytogenetic nomenclature is provided in Fig. 1.

CytoConverter uses the coordinates of cytobands as specified by the cytoband.txt file (resolution 850 bands, the maximum available) from the UCSC Genome Browser R/Builds folder. A web interface was created using Rshiny (Chang et al., 2018 [4]), an R package used to build interactive web applications that run R in the background.

## Results

Here we provide a set of examples demonstrating CytoConverter’s capabilities.

### Single karyotype example

**Fig. 2:**
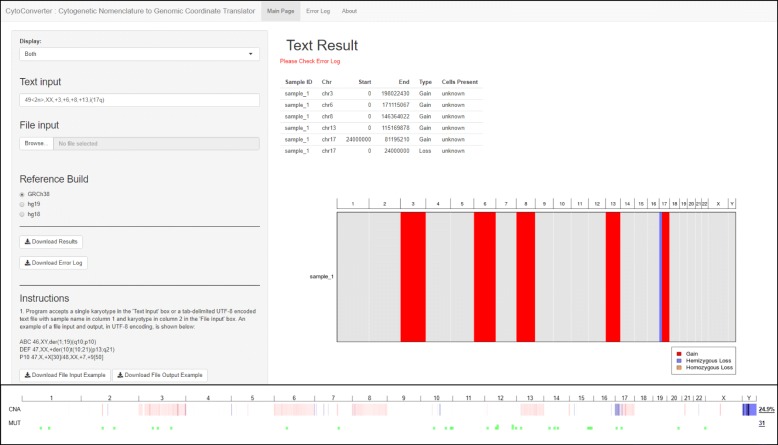
CytoConverter output from a cancer cell line. The top panel shows a screenshot of CytoConverter as applied to the karyotype of the cell line AML-193. The output produced by our web tool is under “Text Result”, wherein the sample is referred to as “sample_1” by default. The bottom panel shows the results from microarray-based copy number analysis of AML-193, as performed in Barretina et al. [1] and visualized using cBioPortal [3, 6]. In cBioPortal output, red indicates gains, blue indicates losses, and a darker color indicates a larger deviation from normal (i.e. a “deeper” deletion or higher-level amplification). Note that cBioPortal reports Y as having been lost, though the cell line is derived from a male patient with only X chromosomes constitutionally and therefore the Y chromosome was not present in the sample

Figure 2 (top panel) shows an example of the interface and output of CytoConverter, applied to the karyotype of AML-193, a cell line derived from a female acute myeloid leukemia (AML) patient. The karyotype for AML-193 is given as 49 < 2n>,XX,+ 3,+ 6,+ 8,+ 13,i (17q) by the Leibniz Institute DSMZ- German Collection of Microorganisms and Cell Cultures (https://www.dsmz.de/catalogues/details/culture/ACC-549.html). As part of the Cancer Cell Line Encyclopedia [1], AML-193 was subjected to microarray-based copy number analysis, the results of which are shown in Fig. 2 (bottom panel). As shown, the copy number changes inferred by CytoConverter from the karyotype match well with the copy number lesions revealed from arrays, except for the more focal changes that are below the limited resolution available on the karyotype level. In this example, the number of cells present are not indicated in the karyotype, but CytoConverter is set up to accommodate complex karyotypes wherein different cells harbor different chromosomal aberrations (see *Example involving multiple clones* subsection below).

### Multiple karyotype example

**Fig. 3:**
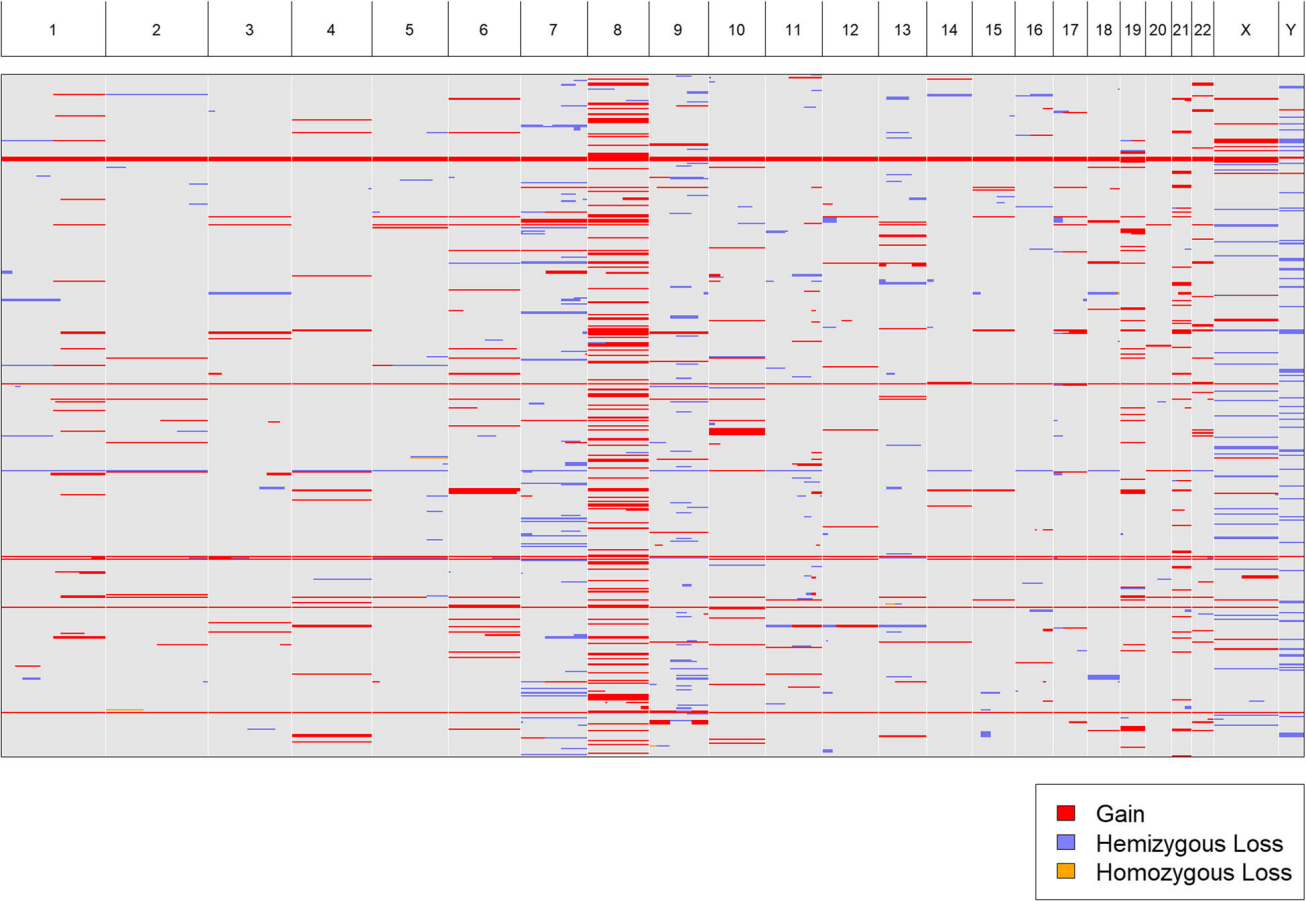
Graphical output by CytoConverter derived from karyotypes of 943 patients from the TARGET consortium [10]

We acquired karyotypes for 943 pediatric patients from the TARGET (“Therapeutically Applicable Research To Generate Effective Treatments”; [10]) project. We uploaded a text, tab-delimited file (Additional file 2: Table S1) containing all 943 karyotypes to CytoConverter to automatically extract the implied gains and losses. CytoConverter’s tabular output is given in Additional file 3: Table S2. Recurrent duplication of chromosome 8 is apparent in CytoConverter’s graphical representation (Fig. 3). In general, it is expected the graphical output will be useful for visual detection of frequent loss or gain of specific chromosomal loci in associated samples.

### Example involving multiple clones

**Fig. 4:**
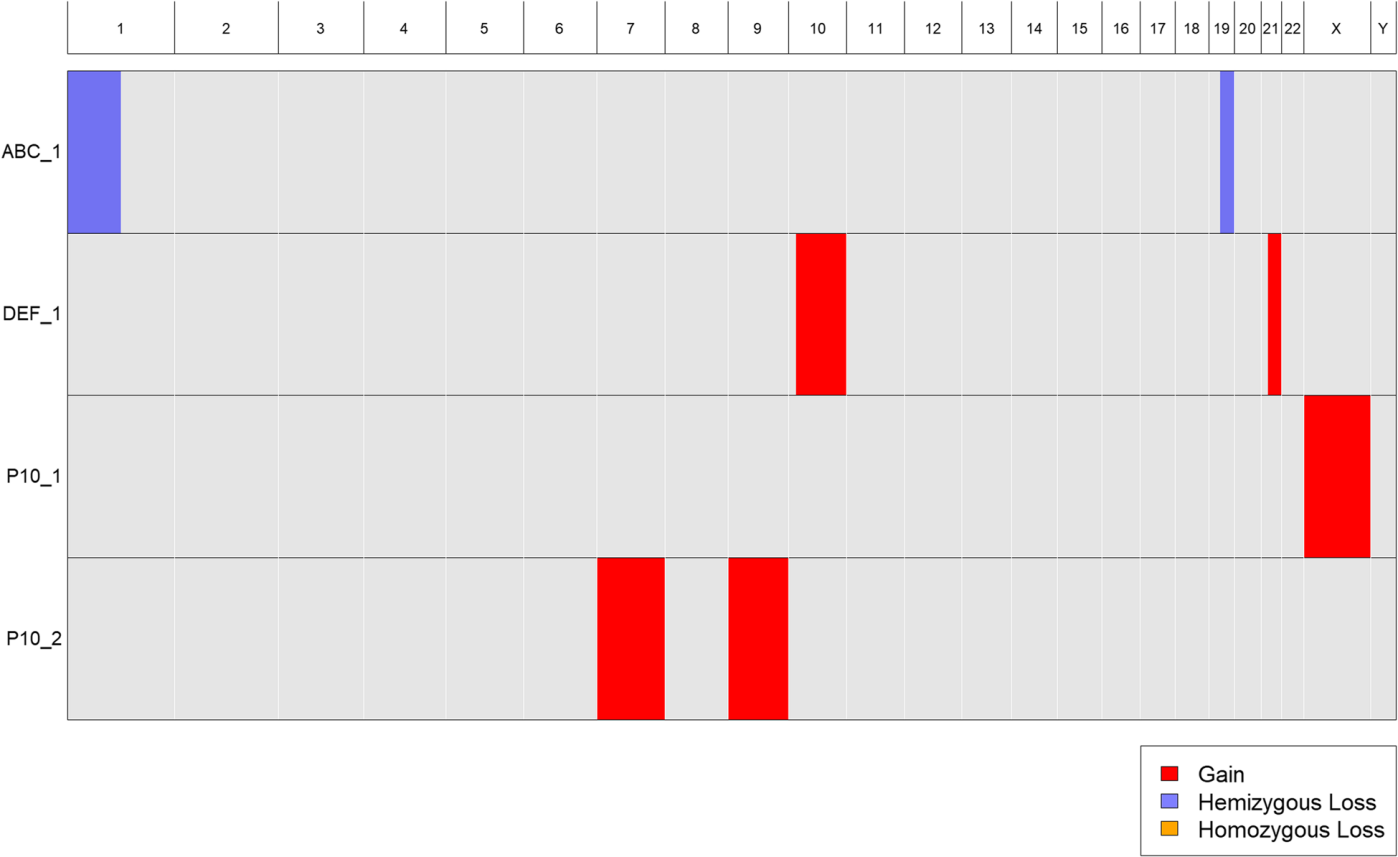
CytoConverter’s graphical output from Table 1 input

Standard cytogenetic nomenclature can accommodate samples with multiple distinct clones, each having a different karyotype. The nomenclature can also be used to indicate the number of visible cells corresponding to each clone karyotype. For example, 47,X,+X [30]/46,XX,+ 7,+ 9[50] indicates a sample with two clones, one with 30 visible cells harboring an extra copy of the X chromosome, and the other with 50 visible cells harboring extra copies of chromosomes 7 and 9. As noted above, CytoConverter handles multiple clones by appending “_n” to the sample name for its *n*^th^ clone’s karyotype. Table 1 gives example input for three patient samples, the last of which has the two-clone karyotype 47,X,+X [30]/46,XX,+ 7,+ 9[50]. CytoConverter’s tabular and graphical output are shown in Table 2 and Fig. 4, respectively.

**Table 1:** Example input to CytoConverter

Sample Name and Karyotype	
ABC	46,XY,der(1;19)(q10;p10)
DEF	47,XX,+der(10)t(10;21)(p13;q21)
P_10	47,X,+X[30]/48,XX,+ 7,+ 9 [50]

**Table 2:** CytoConverter’s tabular output from input given in Table 1

Sample ID	Chr	Start	End	Type	Cells Present
ABC_1	chr1	0	125,000,000	Loss	unknown
ABC_1	chr19	26,500,000	59,128,983	Loss	unknown
DEF_1	chr10	12,200,000	135,534,747	Gain	unknown
DEF_1	chr21	16,400,000	48,129,895	Gain	unknown
P_10_1	chrX	0	155,270,560	Gain	30 of 80
P_10_2	chr7	0	159,138,663	Gain	50 of 80
P_10_2	chr9	0	141,213,431	Gain	50 of 80

### Additional cytogenetic lesions and examples

There is a substantial diversity of cytogenetic terms that CytoConverter is able to handle beyond those shown above. We detail CytoConverter’s approach to parsing each of these in the Additional file 1, where we also provide results from its parsing of several more example karyotypes.

## Conclusions

In summary, we have developed a user-friendly web-based tool that allows users to input any number of human karyotypes, and obtain the genomic coordinates of all gains and losses implied by each of the karyotypes. We anticipate that this tool will be of considerable value to the community for analyzing archival patient samples, as well as samples for which higher-resolution copy number data is unavailable. It should be noted that CytoConverter only reports net gain and loss of chromosomal material relative to the normal, diploid 2n. In keeping with the standards for array-based copy number inference, we do not report, for example, balanced translocations or inversions. This is an area for future development.

## Availability and requirements

Project name: CytoConverter.

Project home page: https://jxw773.shinyapps.io/Cytogenetic__software/

Operating system(s): Platform independent.

Programming language: R.

Other requirements: R 3.5.3 or higher.

License: GNU v.3.0.

Any restrictions to use by non-academics: None.

## Additional files

Additional file 1: Additional material provided describing uses and caveats of CytoConverter including example input. (DOCX 136 kb)

Additional file 2: Sample table containing karyotypes for 943 pediatric patients from the TARGET (“Therapeutically Applicable Research To Generate Effective Treatments”; [10]) project. (TXT 54 kb)

Additional file 3: Table output generated by CytoConverter from sample input from Additional file 2. (TXT 56 kb)

## Data Availability

Some data analyzed was derived from cBioPortal from the CCLE cell line data. https://www.cbioportal.org/patient?studyId=cellline_ccle_broad&caseId=AML-193. Karyotype was derived from https://www.dsmz.de/catalogues/details/culture/ACC-549.html

## Abbreviations

AML: Acute myeloid leukemia
ISCN: International System for Human Cytogenomic Nomenclature
NCBI: National Center for Biotechnology Information
NGS: Next-generation sequencing
