# Supplementary material for: CytoConverter: a web-based tool to convert karyotypes to genomic coordinates
Source: BMC Bioinformatics. 2019 Sep 11;20:467. doi: 10.1186/s12859-019-3062-4 (PMC6739950; doi:10.1186/s12859-019-3062-4)
Supplement: Supplementary file 1 — Additional material provided describing uses and caveats of CytoConverter including example input. (DOCX 136 kb) [file 12859_2019_3062_MOESM1_ESM.docx]

**Supplementary Material**

**Introduction**

CytoConverter is a program that extracts the net losses and gains from a karyotype written in cytogenetic nomenclature. The results are given in genomic coordinates according to GRCh38.The program is available as a website and as a script. Instructions for website use are on the website. Encoding of the input and output is in UTF-8 as per Rshiny requirements; certain characters, such as the ~ may not convert properly if a different encoding was used. This applies to the website only.

To run CytoConverter in R, download the R script CytoConverter.R from the website and source it in an R environment. This will make available a function called CytoConverter which takes as input a character string or a two-column data frame with no header, with the first column containing the sample name and second containing the karyotype. Sample names must be unique.

CytoConverter divides a sample into individual clones and reports results according to each clone. It will return a table with the sample name (adding “_n” to the sample name for gains/losses present in the nth clone in the karyotype, if multiple clones are present), beginning coordinate, ending coordinate, whether the region was lost or gained, and number of cells present of a clone out of total number of cells in a sample with that gain or loss, if that information is provided in the input karyotype. For composite karyotypes, the number will be a range from one cell to the number of cells counted for the composite karyotype. If CytoConverter returns no output, then this indicates that the input karyotype was either incorrect, or there were neither gains nor losses.

CytoConverter uses the coordinates of cytobands as specified by the cytoband.txt file (resolution 850 bands, the maximum available) from the UCSC Genome Browser, build GRCh38/hg38 to hg18

Karyotypes from FISH, microarrays, region-specific assays (RSAs), and sequence-based assays cannot be processed by the program due to the different nomenclature used. Abnormal chromosomes labeled with “?” are not supported, as question marks indicate that information in the karyotype is unknown. When tildas (~) are used to indicate range, the first band listed is always used.

**CytoConverter’s handing of various cytogenetic nomenclature features**

**Conversion from cytobands to genomic coordinates**

CytoConverter makes use of the cytoBand.txt table, downloaded from UCSC Genome Browser website. For any cytoband present in an input karyotype, CytoConverter queries the table to convert the band into GRCh38 coordinate. The resolution given by the karyotype is used to determine the range of any chromosomal aberration. For example, if the karyotype indicates that a deletion spans from 1p35 to 1p32, then the genomic coordinates output by CytoConverter will span the coordinates corresponding to cytobands p35.3 and p32.1. If q10 is indicated, it is assumed that the lesion extends to the centromere, and therefore the centromere’s coordinate is obtained from the table and used.

**Long form vs. short form**

Cytogenetic nomenclature has two broad forms for describing chromosome aberrations. The less common method is using long form. Long form describes the entire chromosome from beginning to end, using a colon (:) to indicate breaks and joins, and -> to indicate a range of bands from the start point to the end point. This is commonly used for chromosomes too difficult to describe through short form. For example, if a derivative chromosome consists of three translocations, a person might use long form to describe the entire chromosome instead.

Short form is the more common method to describe aberrations. In short form, the nature of the aberration, such as deletion or a translocation is indicated, and then the affected area of the aberration is indicated by the bands flanking the region. If only one band is present, that indicates that the aberration extends from the end of the arm to the band.

**Whole chromosome additions/deletions**

*Example:* +7, -9

If a whole chromosome has been added or deleted, CytoConverter outputs the genomic coordinates of the entire chromosome and labels either a gain or a loss.

**Polyploidy/haploidy**

If polyploidy is explicitly stated:

*Example:* 69<3n>,XXX

If a polyploidy is explicitly stated in angle brackets following the chromosome number, CytoConverter computes the number of sets of autosomes gained as the difference between the number inside the brackets and 2n (diploidy).

If polyploidy is implied:

*Example:* 69,XXX

The change in total chromosome number that is due to autosome aberrations is counted, subtracted from the number of chromosomes in the karyotype and divided by 22. If this value is positive, the value describes the additional sets of autosomes present beyond diploidy. If the value is -1, there is a haploidy and half of the autosomes were removed. Sex chromosomes are counted by themselves and not in this count.

**Constitutional sex chromosome number**

*Example:* 70,XXX,+8

Sex chromosomes are handled differently than autosomes. If a Y chromosome is present, CytoConverter decides whether to compare the sex chromosome number to XX or XY. If there is no Y, the normal number of X chromosomes is 2 and normal number of Y chromosomes is 0. If there is a Y, the normal numbers of X and Y chromosomes are each 1. The numbers of unmodified sex chromosomes, which follows the first number in the karyotype (number of chromosomes), are counted and stored. The numbers of sex chromosome added, deleted, and modified are tracked throughout the karyotype. These values are compiled and compared to the normal number of X and Y chromosomes, and any additions or deletions are output if present.

**Deletions**

*Example:* del(1)(q21)

Section deleted is translated into genomic coordinates and marked as a loss.

**Duplications/triplications/quadruplications/tandem duplications**

*Examples:* dup(1)(q21)

trp(1)(q21)

qdq(1)(q21)

tan(1)(q21)

Section that is duplicated/triplicated/quadruplicated is translated into genomic coordinates and marked as a gain.

**Translocations**

*Example:* t(12;16)(p13;p11.1)

t(9;22;17)(q34;q11.2;q22)

Any translocations present in the karyotype are recorded in a table and translated into their resultant chromosomes’ cytobands in case they are later referenced in derivative chromosomes. Note that balanced translocations imply no net gain or loss of chromosome material and therefore have no effect on CytoConverter’s output.

ISCN nomenclature allows referencing of an abbreviated translocation later within the same karyotype. For example, the translocation t(9;11)(q10;q10) may be referenced simply as t(9;11) later within the same karyotype. CytoConverter can accommodate this even between different clones. However, an abbreviated karyotype may not be referenced between different patient samples (we have observed such misuse of nomenclature in published paper supplements). If there are two different translocations involving the same set of chromosomes, only one of the translocations should use the abbreviated form. For instance, if there are two translocations between chromosome 5 and 6, please use the abbreviation t(5;6) for only one of the translocations.

**Insertions**

*Example:* ins(5;2)(p14;q22q32)

ins(2)(q13p23p13)

Any insertions that are detected are recorded in a table of its resultant chromosomes in case they are referenced later in a derivative chromosome. In ISCN nomenclature, insertions that are not derivative chromosomes are balanced and therefore do not affect CytoConverter’s output. See Translocations subsection immediately above for rules regarding abbreviations in general, which also apply here to Insertions.

**Isochromosomes**

*Examples:* i(8)(q10)

i(8q)

An isochromosome is a chromosome that consists of a duplicated arm such that the chromosome is symmetrical. For example, i(8)(q10) consists of two q arms of chromosome 8.

CytoConverter reports the genomic coordinates corresponding to the arm indicated as being gained, and those corresponding to the arm not indicated as being lost.

**Dicentric/Pseudodicentric chromosome/Tricentric chromosomes/Isodicentric chromosome**

*Examples:* dic(10;21)(p10;q10)

psu dic(12;21)(12qter->12p11.2::21p11.2->21qter)

trc(10;14;21)(p10;p11.2q12;q10)

idic(21)(q22.3)

A dicentric chromosome is a chromosome formed from the combining of two chromosomes that results in a chromosome with two centromeres. A pseudodicentric chromosome is a dicentric chromosome in which only one of the centromeres is active, but two still exist on the chromosome. A tricentric chromosome is a chromosome formed from combining three chromosomes that result in a chromosome with three centromeres. An isodicentric chromosome is a chromosome that is symmetrical and the result of two of the same chromosomes merging.

The manner in which these chromosomes are parsed varies depending on if the nomenclature is written in long form or short form. In long form, the complements of the regions indicated in the karyotype are marked as a loss. For short form, CytoConverter marks the regions indicated in the karyotype as a loss except in the case of tricentric chromosomes. For tricentric chromosomes, the regions indicated in the outer two chromosomes are marked as a loss and the complement of the region in the middle is marked as a loss. For example, for dic(10;21)(p10;q10), CytoConverter marks the p10 region of chromosome 10 and the q10 region as a loss.

**Ring chromosomes**

*Examples:* r(7)(p22q36)

+r

This type of aberration may consist of one or more chromosomes.

The complement of the region indicated is lost, so CytoConverter determines the genomic coordinates of the complement and reports it as being lost. Rings that do not indicate chromosome numbers involved (e.g. +r) are ignored except for counting chromosomes to calculate polyploidy.

**Derivative chromosomes/Recombinant chromosomes**

*Examples:* der(9)del(9)(p12)del(9)(q31)

der(9)(:p12->q31:)

rec(9)(p12)

A derivative chromosome is a chromosome that harbors more than one aberration and/or is derived from a translocation and/or an insertion. Recombinant chromosomes are chromosomes that are rearranged during meiosis and are, for CytoConverter, functionally equivalent to derivative chromosomes.

The parsing of derivative chromosomes is an extremely complex processes and is merely summarized in this supplement. CytoConverter parses each individual aberration in a derivative chromosome. If the derivative involves translocations or insertions, CytoConverter generates and references a table with translocations, insertions and their respective derivative chromosomes that each aberration describes and retrieves the derivative chromosome indicated. Any chromosome aberrations that are not indicated in the der() field that are present are marked as a gain; any areas not present in the derivative chromosome corresponding to the chromosome(s) indicated in the der() field are marked as a loss. For example, for a derivative chromosome der(9)t(9;11)(q10:q10), the chromosome indicated is a derivative chromosome that consists of the p arm of chromosome 9 and the q arm of chromosome 11. Because the chromosome indicated in the first field is 9, the q arm of chromosome 9 is marked as a loss and the q arm of chromosome 10, not indicated under the der field, is marked as a gain in material.

Since the breadth of possible aberrations under the derivative chromosome label is very wide, here we describe certain examples that exemplify subtypes of the use of derivative chromosomes and how CytoConverter handles them:

- der(1;19)(q10;p10)

This derivative chromosome consists of the q arm of chromosome 1 and the p arm of chromosome 19. CytoConverter will mark the p arm of chromosome 1 and the q arm of chromosome 19 as losses (the complement of the indicated regions).

- der(6)(qter->q25.2::p22.2->pter)

This derivative chromosome consists of the ranges described in long form. CytoConverter converts all the ranges present in the chromosome to genomic coordinates. The complement of these ranges is marked as a loss.

- der(16)t(8;16)(q10;p11)

This derivative chromosome is derived from a translocation between chromosome 8 and 16. CytoConverter will reference a table to find the translocation marked in the table and then find the relevant derivative chromosome (chromosome 16) and parse that chromosome. The end result is that CytoConverter will mark the region on chromosome 16 from p11 to the end of the p arm as a loss, and the q arm on chromosome 8 as a gain. The reason there is a reference table to update is so that karyotypes such as 47, t(8;16)(q10;p11), +der(16)t(8;16) can be parsed correctly.

- der(1)ins(1;7)(q21;p11.2p21)

This chromosome is derived from an insertion. CytoConverter will look up its reference table to find the insertion, and obtain the derivative chromosome 1 of that insertion. The end result is that CytoConverter will mark the section inserted from chromosome 7 as a gain.

- der(9)del(9)(p12)t(9;13)(q34;q11)

This is a derivative chromosome consisting of both a deletion and a translocation. Each aberration will be parsed individually. At the end, any inconsistencies that result are either removed or fixed (inconsistencies here would be overlap between the two genomic ranges).

**Robertsonian chromosome**

*Example:* rob(13;21)(q10;q10)

A Robertsonian chromosome is a type of derivative chromosome resulting from translocations that are with respect to acrocentric chromosomes. The complement of the region indicated is lost, and CytoConverter determines the genomic coordinates of the complement and reports it as being lost.

**Isoderivative chromosomes**

*Example:* ider(22)(q10)t(9;22)(q34;q11.2)

An isoderivative chromosome is a derivative chromosome comprised of two copies of one derivative chromosome arm, fused at a centromere. The same processes for parsing derivative chromosomes is used except that any gains on the isoderivative chromosome arm are doubled during processing before net gains and losses are calculated. The other arm of the main chromosome(s) is marked as a loss.

**Additional chromosomes**

*Example:* +der(10)t(10;21)(p13;q21)

+del(13)(q12q22)

In ISCN nomenclature, a ‘+’ before a chromosome indicates that that chromosome is present in the cell in addition to the normal number of chromosomes. It follows that losses indicated in the additional chromosome will not be counted as such, but all chromosomal regions present in the additional chromosome will be counted as a gain. For example, for +del(13)(q12q22), everything except the range from band q12 to band q22 would be counted as a gain.

**Inversions**

*Example*: inv(3)(q21q26.2)

Inversions are ignored since there is no net gain or loss of chromosomal material.

**Additional material**

*Example*: add(19)(p13.3)

Since these are sections of a chromosome where additional material of unidentified origin is present, these are ignored.

**Marker chromosome**

*Example***:** +mar

These are chromosomes present whose origin is undetermined. These are ignored except in counting chromosome number for calculating polyploidy.

**Clonal evolution**

*Example*: 26,X,+4,+6,+21 [3]/52,idemx2

46,XY,t(9;22)(q34;q11.2)[3]/92,slX2[5]/93,sdl, +8 [2]

Clonal evolution occurs when derivative clones evolve from an initial cell. The derivative clones are called subclones. If all subclones evolved from the mainline (first one listed in karyotype), the term idem is often used to indicate this fact. However, sidelines also occur that may evolve from subsequent subclones and such lines are denoted sl/sdl (evolved from mainline) sdl1 (evolved from first subclone) sdl2 (evolved from second subclone), etc. Currently, when the sl system is used, the assumption is that every sideline has evolved from the sideline preceding it, unless sl is indicated, which the program interprets as having evolved from the first clone line. However, if the need arises, the ability to have more customization in interpreting sidelines may be coded in in future versions of CytoConverter.

**More Complicated Examples**

Here we give several examples of CytoConverter output for more complex input karyotypes.

*Input:*

130<6n>XXX, -Y, -Y, -Y,inc,add(1)(p22),i(8q),i(?5q)x2,del(6)(q23),t(14;15)(p10;q10), der(19)t(1;19)(q24;q13)

*Output:*

| Sample ID | Chr | Start | End | Type | Cells Present |
| --- | --- | --- | --- | --- | --- |
| sample_1 | chr1 | 0 | 249250621 | Gain | unknown |
| sample_1 | chr10 | 0 | 135534747 | Gain | unknown |
| sample_1 | chr11 | 0 | 135006516 | Gain | unknown |
| sample_1 | chr12 | 0 | 133851895 | Gain | unknown |
| sample_1 | chr13 | 0 | 115169878 | Gain | unknown |
| sample_1 | chr14 | 0 | 107349540 | Gain | unknown |
| sample_1 | chr15 | 0 | 102531392 | Gain | unknown |
| sample_1 | chr16 | 0 | 90354753 | Gain | unknown |
| sample_1 | chr17 | 0 | 81195210 | Gain | unknown |
| sample_1 | chr18 | 0 | 78077248 | Gain | unknown |
| sample_1 | chr19 | 0 | 59128983 | Gain | unknown |
| sample_1 | chr2 | 0 | 243199373 | Gain | unknown |
| sample_1 | chr20 | 0 | 63025520 | Gain | unknown |
| sample_1 | chr21 | 0 | 48129895 | Gain | unknown |
| sample_1 | chr22 | 0 | 51304566 | Gain | unknown |
| sample_1 | chr3 | 0 | 198022430 | Gain | unknown |
| sample_1 | chr4 | 0 | 191154276 | Gain | unknown |
| sample_1 | chr5 | 0 | 180915260 | Gain | unknown |
| sample_1 | chr6 | 0 | 171115067 | Gain | unknown |
| sample_1 | chr7 | 0 | 159138663 | Gain | unknown |
| sample_1 | chr8 | 0 | 146364022 | Gain | unknown |
| sample_1 | chr9 | 0 | 141213431 | Gain | unknown |
| sample_1 | chrX | 0 | 155270560 | Gain | unknown |
| sample_1 | chrY | 0 | 59373566 | Loss | unknown |
| sample_1 | chrY | 0 | 59373566 | Loss | unknown |
| sample_1 | chrY | 0 | 59373566 | Loss | unknown |

*
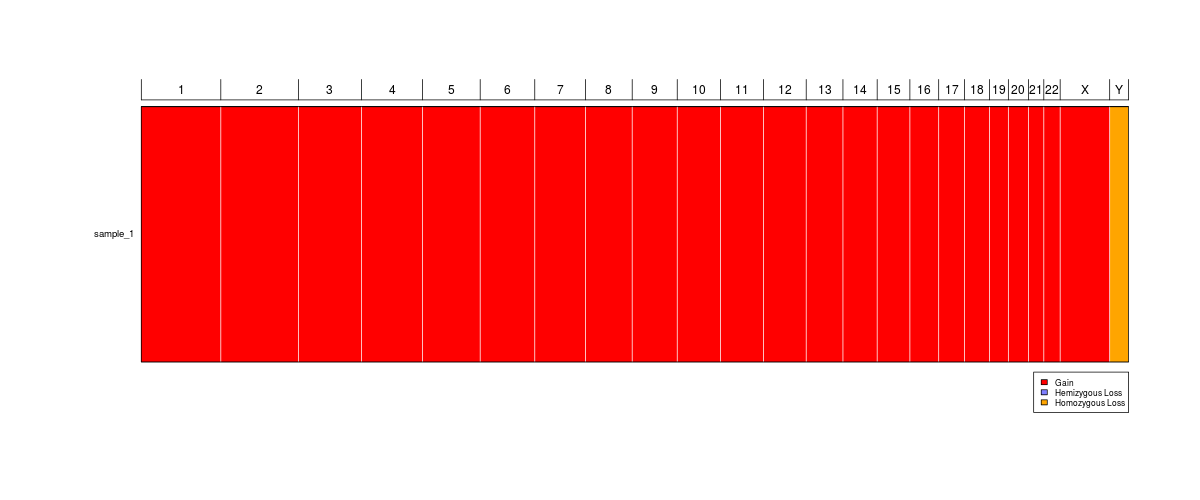
*

*Input:*

40-50,XX,der(1;14)(q10;q10),+der(16)t(8;16)(q10;p11)X2,+mar,+2mar2

*Output:*

| Sample ID | Chr | Start | End | Type | Cells Present |
| --- | --- | --- | --- | --- | --- |
| sample_1 | chr16 | 34600000 | 90354753 | Gain | unknown |
| sample_1 | chr8 | 45600000 | 146364022 | Gain | unknown |
| sample_1 | chr1 | 0 | 125000000 | Loss | unknown |
| sample_1 | chr14 | 0 | 17600000 | Loss | unknown |


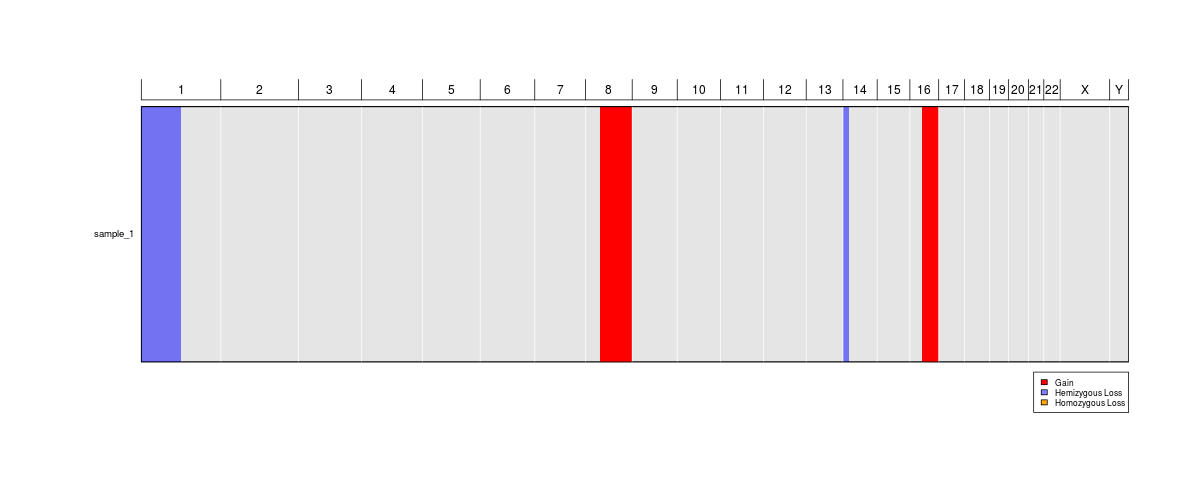


*Input:*

46,XX,der(1)t(1;11)(p32;q13)t(1;3)(q25;q21)

*Output:*

| Sample ID | Chr | Start | End | Type | Cells Present |
| --- | --- | --- | --- | --- | --- |
| sample_1 | chr11 | 63400000 | 135006516 | Gain | unknown |
| sample_1 | chr3 | 121900000 | 198022430 | Gain | unknown |
| sample_1 | chr1 | 0 | 61300000 | Loss | unknown |
| sample_1 | chr1 | 172900000 | 249250621 | Loss | unknown |


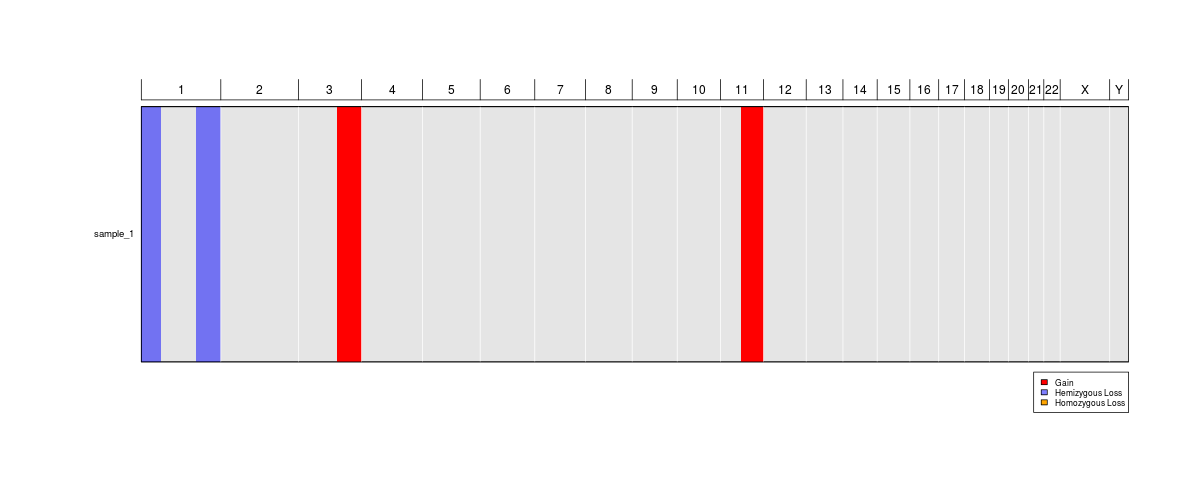


*Input:*

46,XX,der(9)del(9)(p12)t(9;13)(q34;q11)

*Output:*

| Sample ID | Chr | Start | End | Type | Cells Present |
| --- | --- | --- | --- | --- | --- |
| sample_1 | chr13 | 17900000 | 115169878 | Gain | unknown |
| sample_1 | chr9 | 0 | 43600000 | Loss | unknown |
| sample_1 | chr9 | 133500000 | 141213431 | Loss | unknown |


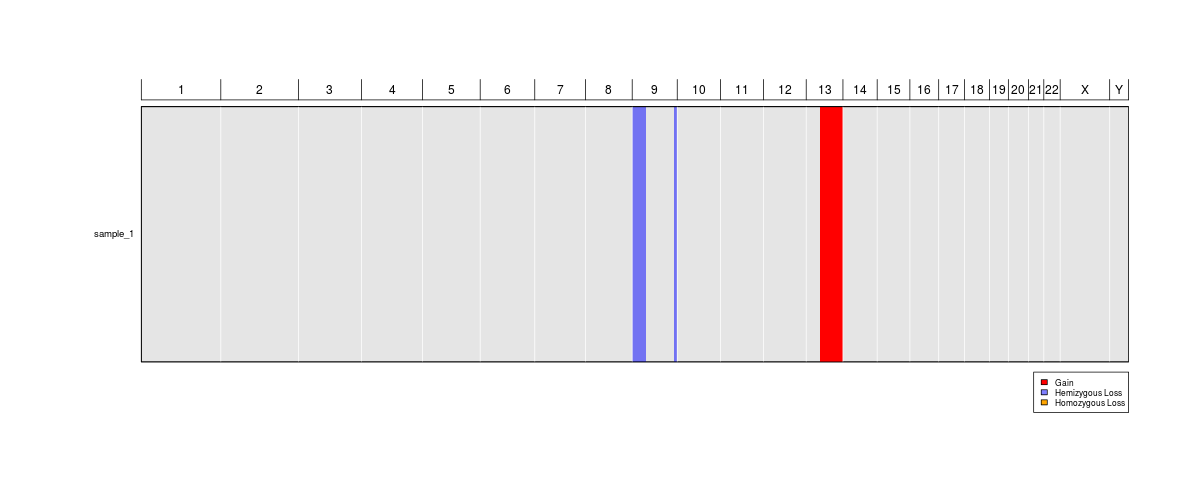


*Input:*

46,XX,ider(22)(q10)t(9;22)(q34;q12)

*Output:*

| Sample ID | Chr | Start | End | Type | Cells Present |
| --- | --- | --- | --- | --- | --- |
| sample_1 | chr22 | 14700000 | 25900000 | Gain | unknown |
| sample_1 | chr22 | 0 | 14700000 | Loss | unknown |
| sample_1 | chr22 | 25900000 | 51304566 | Loss | unknown |
| sample_1 | chr9 | 130300000 | 141213431 | Gain | unknown |


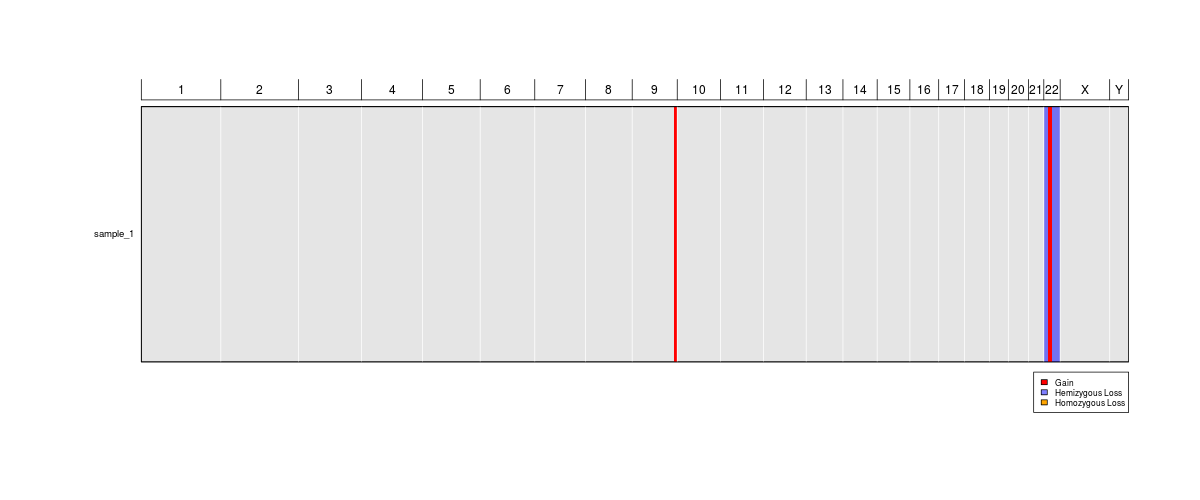


*Input:*

47,XX,+del(13)(q12q22)

*Output:*

| Sample ID | Chr | Start | End | Type | Cells Present |
| --- | --- | --- | --- | --- | --- |
| sample_1 | chr13 | 0 | 19500000 | Gain | unknown |
| sample_1 | chr13 | 79000000 | 115169878 | Gain | unknown |


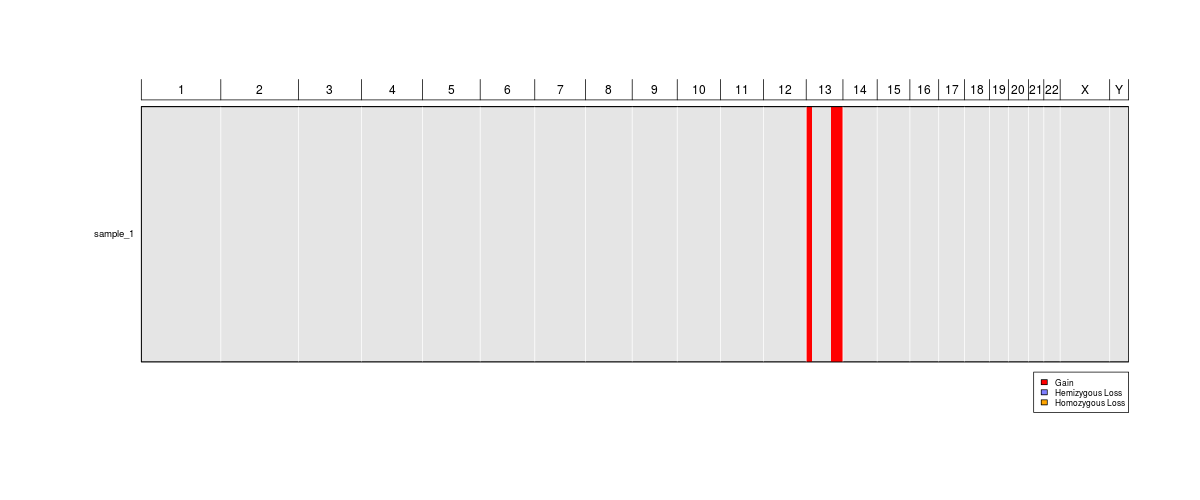


*Input:*

46,XX,+der(10)t(10;21)(p13;q21)

*Output:*

| Sample ID | Chr | Start | End | Type | Cells Present |
| --- | --- | --- | --- | --- | --- |
| sample_1 | chr10 | 12200000 | 135534747 | Gain | unknown |
| sample_1 | chr21 | 16400000 | 48129895 | Gain | unknown |


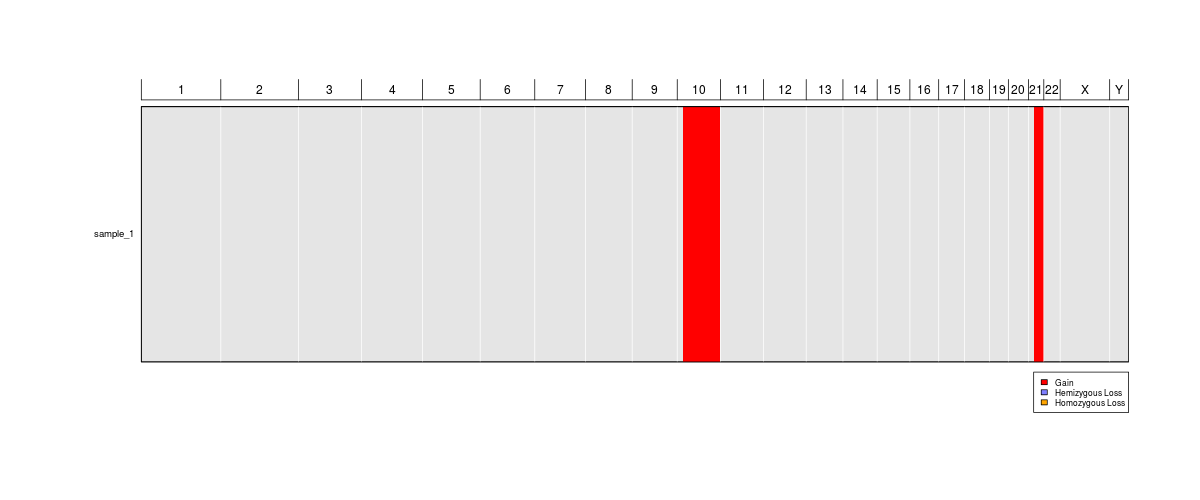


*Input:*

46,XY,t(9;22)(q34;q11.2)[3]/92,slX2[5]/93,sdl,+8[2]

*Output:*

| Sample ID | Chr | Start | End | Type | Cells Present |
| --- | --- | --- | --- | --- | --- |
| sample_2 | chrX | 0 | 155270560 | Gain | 5 of 10 |
| sample_2 | chrY | 0 | 59373566 | Gain | 5 of 10 |
| sample_2 | chr1 | 0 | 249250621 | Gain | 5 of 10 |
| sample_2 | chr2 | 0 | 243199373 | Gain | 5 of 10 |
| sample_2 | chr3 | 0 | 198022430 | Gain | 5 of 10 |
| sample_2 | chr4 | 0 | 191154276 | Gain | 5 of 10 |
| sample_2 | chr5 | 0 | 180915260 | Gain | 5 of 10 |
| sample_2 | chr6 | 0 | 171115067 | Gain | 5 of 10 |
| sample_2 | chr7 | 0 | 159138663 | Gain | 5 of 10 |
| sample_2 | chr8 | 0 | 146364022 | Gain | 5 of 10 |
| sample_2 | chr9 | 0 | 141213431 | Gain | 5 of 10 |
| sample_2 | chr10 | 0 | 135534747 | Gain | 5 of 10 |
| sample_2 | chr11 | 0 | 135006516 | Gain | 5 of 10 |
| sample_2 | chr12 | 0 | 133851895 | Gain | 5 of 10 |
| sample_2 | chr13 | 0 | 115169878 | Gain | 5 of 10 |
| sample_2 | chr14 | 0 | 107349540 | Gain | 5 of 10 |
| sample_2 | chr15 | 0 | 102531392 | Gain | 5 of 10 |
| sample_2 | chr16 | 0 | 90354753 | Gain | 5 of 10 |
| sample_2 | chr17 | 0 | 81195210 | Gain | 5 of 10 |
| sample_2 | chr18 | 0 | 78077248 | Gain | 5 of 10 |
| sample_2 | chr19 | 0 | 59128983 | Gain | 5 of 10 |
| sample_2 | chr20 | 0 | 63025520 | Gain | 5 of 10 |
| sample_2 | chr21 | 0 | 48129895 | Gain | 5 of 10 |
| sample_2 | chr22 | 0 | 51304566 | Gain | 5 of 10 |
| sample_3 | chr8 | 0 | 146364022 | Gain | 2 of 10 |
| sample_3 | chrX | 0 | 155270560 | Gain | 2 of 10 |
| sample_3 | chrY | 0 | 59373566 | Gain | 2 of 10 |
| sample_3 | chr1 | 0 | 249250621 | Gain | 2 of 10 |
| sample_3 | chr2 | 0 | 243199373 | Gain | 2 of 10 |
| sample_3 | chr3 | 0 | 198022430 | Gain | 2 of 10 |
| sample_3 | chr4 | 0 | 191154276 | Gain | 2 of 10 |
| sample_3 | chr5 | 0 | 180915260 | Gain | 2 of 10 |
| sample_3 | chr6 | 0 | 171115067 | Gain | 2 of 10 |
| sample_3 | chr7 | 0 | 159138663 | Gain | 2 of 10 |
| sample_3 | chr9 | 0 | 141213431 | Gain | 2 of 10 |
| sample_3 | chr10 | 0 | 135534747 | Gain | 2 of 10 |
| sample_3 | chr11 | 0 | 135006516 | Gain | 2 of 10 |
| sample_3 | chr12 | 0 | 133851895 | Gain | 2 of 10 |
| sample_3 | chr13 | 0 | 115169878 | Gain | 2 of 10 |
| sample_3 | chr14 | 0 | 107349540 | Gain | 2 of 10 |
| sample_3 | chr15 | 0 | 102531392 | Gain | 2 of 10 |
| sample_3 | chr16 | 0 | 90354753 | Gain | 2 of 10 |
| sample_3 | chr17 | 0 | 81195210 | Gain | 2 of 10 |
| sample_3 | chr18 | 0 | 78077248 | Gain | 2 of 10 |
| sample_3 | chr19 | 0 | 59128983 | Gain | 2 of 10 |
| sample_3 | chr20 | 0 | 63025520 | Gain | 2 of 10 |
| sample_3 | chr21 | 0 | 48129895 | Gain | 2 of 10 |
| sample_3 | chr22 | 0 | 51304566 | Gain | 2 of 10 |

*
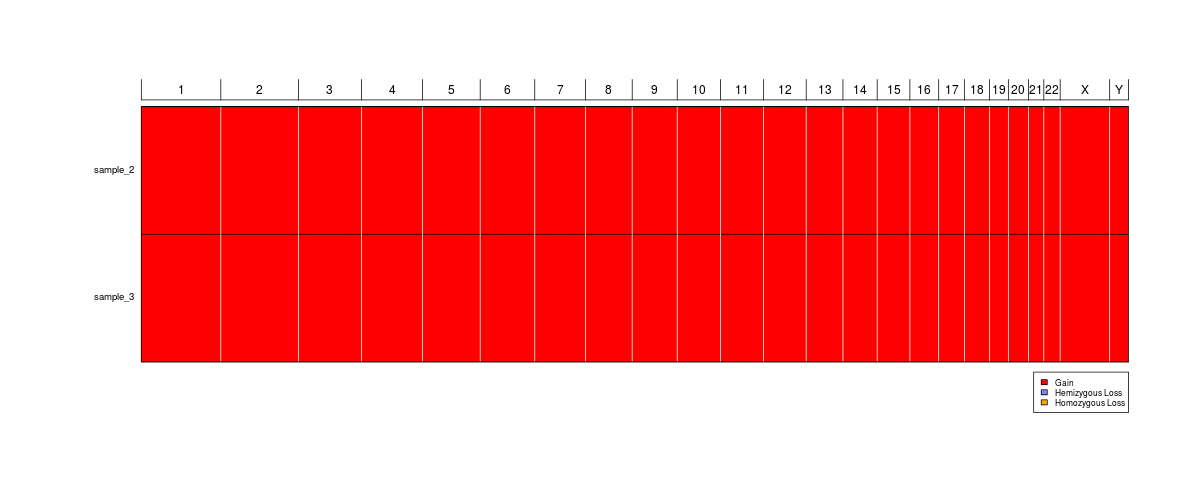
*

**Known issues**

- Derivative chromosomes involving translocations of the same chromosome number (or sex chromosome) will generate an error. In the ISCN 2016, the relevant chromosome is denoted by underlining; however, underlining is not supported in plain text files or in the R.
- The counting of chromosomes does not work well when question marks are present in the karyotypes or the karyotypes do not follow the ISCN. Haploidy or polyploidy is to be interpreted with caution under such circumstances.
- CytoConverter will not processes karyotypes such as 48,XX,del(8)(p11),dup(8)(q23)x2 properly because an overall count of aberrations per chromosomes is not kept as of now. Please input the karyotype as 48,XX,+del(8)(p11),dup(8)(q23)x2 instead.
- The program allows for some deviations from the ISCN 2016 to be processed correctly but please don’t push it. For instance, 46XX,+7 will be interpreted correctly, as will using – in place of ~. The program is not case sensitive, and errors in spacing are allowed.
